# Infant Caudate Lobe Injury With Retroperitoneal Hematoma: A Case Report

**DOI:** 10.7759/cureus.27327

**Published:** 2022-07-27

**Authors:** Toshiro Imamoto, Makoto Sawano, Ikuya Ueta

**Affiliations:** 1 Department of Emergency Medicine and Critical Care, Saitama Medical Center, Saitama Medical University, Kawagoe, JPN; 2 Pediatric Critical Care Medicine, Saitama Children's Medical Center, Saitama, JPN

**Keywords:** pre-operative management, pediatric trauma, retroperitoneal hematoma, caudate lobe injury, case report

## Abstract

Liver injury, especially caudate lobe injury, is an extremely rare form of injury in infants. In most cases, liver injury results in intraperitoneal hemorrhage when the capsule is ruptured, and circulatory dynamics deteriorate early. Caudate lobe injuries, however, often present with a high retroperitoneal hematoma. The diagnosis is difficult to identify with a focused assessment with sonography for trauma (FAST) in the initial treatment of trauma and may even be delayed without contrast-enhanced CT imaging. A one-month-old postoperative boy with congenital heart disease was involved in a motor vehicle accident and presented with a single caudate lobe injury. He was not wearing a seatbelt, and it was thought that the caudate lobe was injured due to shearing forces in the cephalocaudal direction at the time of the accident. The patient did not go into shock when he first came to our hospital, but a few hours after admission, he went into shock and required surgical hemostasis. The postoperative course was good, and the patient was discharged alive one month later. The lesson to be learned from this case is that caudate lobe injuries are often associated with retroperitoneal hematoma and slow deterioration of hemodynamics, so it is important not to miss small changes in the child's vitals and to be willing to perform contrast-enhanced CT imaging depending on the type of injury.

## Introduction

The liver is the second most common injured organ in blunt abdominal trauma in childhood after the spleen. However, liver injury in infancy is relatively rare [1]. Further, injury at an almost neonatal age is even rarer [2]. The treatment strategy for liver injury also depends on the type of injury, but cases in which the capsule is disrupted and intra-abdominal bleeding is present can be fatal if not properly diagnosed and treated [3]. A one-month-old boy with postoperative congenital heart disease suffered a liver injury in a road traffic accident and went into shock. We report a case in which the bleeding site of a liver injury was a retroperitoneal hematoma rather than an intraperitoneal hemorrhage, which slowed the course of the injury and saved the patient's life. Because of the low energy mechanism of the injury, the severity of the trauma was initially misjudged, and initial treatment was delayed. We discuss the importance of noticing changes in vital signs in the diagnosis of liver injury in infants and the anatomical sites that are more likely to result in a retroperitoneal hematoma.

## Case presentation

A one-month-old boy whose body weight is 4.4 kg on aspirin for postoperative total anomalous pulmonary venous connection (TAPVC) was placed in an unfastened child seat in the back seat of a car driven by his father. The vehicle crashed into an electric pole in the oncoming lane at a speed of 60 km/h. The child was thrown out of the child seat by the impact. He was transported to the hospital by ambulance. 

On arrival, the child's airway was open and he was crying. Arterial oxygen saturation was stable at 95% on room air. The heart rate was tachycardia from 150 to 200 beats/min when not crying or crying. Abdominal ultrasonography identified damage to the liver parenchyma, but no echo-free space suggestive of intra-abdominal bleeding was apparent. We recognized a laceration in the liver parenchyma on ultrasound at this time but did not recognize that the site was S1 and that the patient presented with a high retroperitoneal hematoma via a lesser sac. On clinical examination, the Glasgow Coma Scale (GCS) was slightly decreased to 13 (E3V4M6). Physical examination revealed bruising on the right anterior forehead, but without other findings. Computed tomography (CT) of the head showed right subarachnoid hemorrhage. The patient was admitted for head and liver injury. Initial blood test results showed an elevated serum lactate level. Prothrombin time-international normalized ratio (PT-INR)** **prolongation and low fibrinogen level and high D-dimer suggest consumptive coagulopathy. Findings of high serum glutamic oxaloacetic transaminase (SGOT) and serum glutamic pyretic transaminase (SGPT) were highly suggestive of liver injury (Table 1).

**Table 1:** Initial blood tests Initial blood test results showed an elevated serum lactate level. PT-INR prolongation and low fibrinogen level and high D-dimer suggest consumptive coagulopathy. Findings of high GOT and GPT were highly suggestive of liver injury.

Initial blood test	Results	Reference range
Lactate (mmol/L)	4.5	0.4-1.6
pH	7.26	7.35-7.45
Hemoglobin (mg/dL)	8.9	9.0-13.5
Platelets (×10000/μL)	34	27-88
Prothrombin time-international normalized ratio (PT-INR)	1.37	1.0-1.2
Activated partial thromboplastin time (APTT; sec)	30	30-45
Fibrinogen (mg/dL）	129	130-330
D-dimer (μg/mL）	59	0-1
Glutamic oxaloacetic transaminase (GOT; U/L）	1247	21-64
Glutamic pyruvate transaminase (GPT; U/L）	2444	201-405
Creatine kinase (CK; U/L）	496	44-315

**Figure 1:**
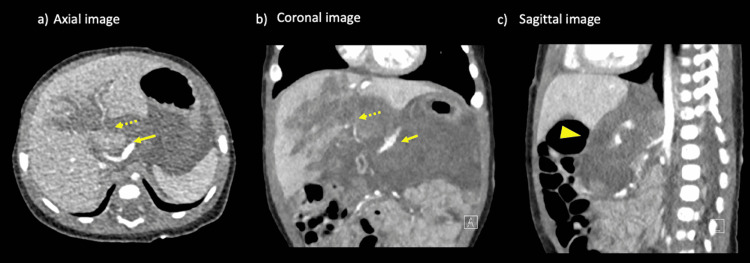
CT of the abdomen A tear in the caudate lobe to the hilar region of the liver is evident along the Cantlie line (dotted arrow). Retroperitoneal hematoma within the hepatic duodenal mesentery, from the anterior surface of the pancreas to the lesser sac (arrowhead). Pseudoaneurysm formation was observed at the site where the celiac artery transitions to the common hepatic artery (arrow).

While waiting for admission, the patient's blood pressure dropped, and systolic blood pressure improved quickly with fluid loading from 50 mmHg to 70 mmHg. Contrast-enhanced CT of the abdomen was performed at this point. CT showed a tear in the caudate lobe along the hilar and Cantlie lines, a retroperitoneal hematoma within the hepatoduodenal mesentery and from the anterior surface of the pancreas to the lesser sac, and the formation of a pseudoaneurysm in the region where the celiac artery transitions to the common hepatic artery, in addition to extravascular leakage in the liver parenchyma. Based on these findings, we determined the American Association for the Surgery of Trauma (AAST) liver injury grade III. However, there is no mention of retroperitoneal hematoma in the current AAST classification, so we judged the depth of the laceration (Figure 1). 

The retrohepatic inferior vena cava showed deformity, but no leakage of contrast medium from the surrounding area was evident. 

**Figure 2:**
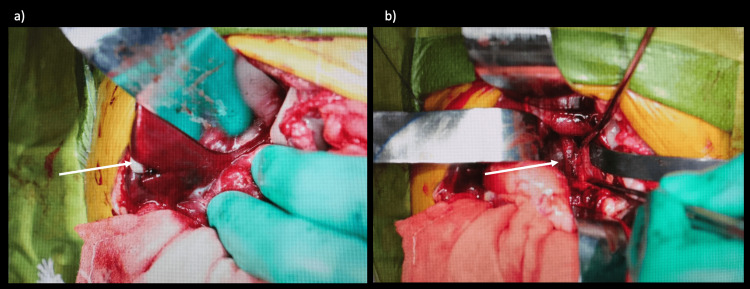
Intraoperative images a) Arterial hemorrhage from the lacerated area of the caudate lobe was observed (arrow). b) Persistent bleeding was also observed from the damaged area of the arterial vascular sheath near the transition from the celiac artery to the common hepatic artery (arrow).

After admission to the pediatric intensive care unit, the patient's blood pressure decreased again. The lowest systolic pressure at this time was 35-40 mmHg. Blood pressure improved with red blood cells administered at 10 mL/kg pumping infusion with a syringe for hypotension; Hb decreased from 8.9 mg/dL initially to 4 mg/dL but improved to 7 mg/dL before surgery. Hemostasis by laparotomy was chosen as the treatment strategy. At the time of laparotomy, little intraperitoneal bleeding was evident. The lesser sac was opened, and a pulsating hematoma was identified in the retroperitoneum. Active bleeding from the caudate lobe was noted. The injured area was sutured using compression, soft coagulation, and TachoSil® to stop the bleeding. Persistent bleeding was also observed from the injured area of the arterial vascular sheath near the transition from the celiac artery to the common hepatic artery (Figure 2).

This bleeding was stopped using a TachoSil® patch. A drain was placed, and the surgery was completed. Intraoperatively, bleeding was 280 mL. As a result, 250 mL of red blood cells, 180 mL of fresh frozen plasma, and 130 mL of platelet concentrates were administered perioperatively. One week after the surgery, the hematoma was confirmed to have shrunken, and oral intake was resumed. The patient was discharged home one month after the surgery. 

## Discussion

Our case suggests that the diagnosis by ultrasound is difficult and requires caution because caudate lobe injuries in children themselves are rare and often present with a clinical picture of retroperitoneal hematoma rather than intraperitoneal hemorrhage. The caudate lobe is an anatomically notable region as a segment that spans the right and left lobes of the liver [4,5].

The lesser omentum is the peritoneum that covers the underside of the liver and continues to the beginning of the duodenum. In other words, this adipose tissue surrounds the caudate lobe of the liver. The lesser sac is the space formed by the greater and lesser omenta and is naturally located posterior to the lesser omentum and anterior to the pancreas, posterior body wall peritoneum, and caudate lobe of the liver. This is not only a finding for adult autopsies but also appears true for children as young as one month old. The caudate lobe is the area of the anterior surface of the inferior vena cava that forms the latest. The caudate lobe can be identified at 9-10 weeks of gestation. Late in pregnancy, rightward rotation of the liver causes the vascular ligament to be positioned anterior to the caudate lobe, and the inferior vena cava forms the posterior border of the caudate lobe [4,5].

Injury to the caudate lobe alone is rare in children [6]. In general, the right lobe of the liver is the most voluminous and the most susceptible to injury due to the proximity of rib fractures and spinal fractures. The left lobe is rather rarely injured, with such cases often caused by direct external force on the upper abdomen. Such injuries can be caused by compression of the thorax or abdomen by the wheels in a motor vehicle accident. Injury to the caudate lobe is very rare and can occur alone, but other areas are often injured at the same time [7]. In the present case, the injury was mainly in the caudate lobe, and a deep laceration to the right lobe was seen along the Cantlie line, as shown by CT. However, since the laceration did not extend to the hepatic surface, a high retroperitoneal hematoma formed due to the anatomy of the caudate lobe, and part of the posterior area of the right lobe faced the retroperitoneal cavity. 

No studies have described caudate lobe injuries; there are only case reports [6,8]. In a case report of a caudate lobe injury, Sacchi et al. reported the anatomical picture of the injury and the fact that the patient's circulation was initially stable but gradually collapsed, requiring surgery. The short hepatic vein and inferior vena cava were torn, and the caudate lobe required sectional resection and direct suture of the inferior vena cava [8]. The clinical picture was very similar to that of the present case. Hemorrhage due to injury to the caudate lobe often flows into the retroperitoneal cavity rather than into the abdominal cavity, resulting in a retroperitoneal hematoma [9,10]. Therefore, due to the limited space compared to the intraperitoneal, the patient may arrive at the hospital in a stable circulatory state immediately after the injury but may gradually enter shock and become severely ill. In the case of injuries to children, especially infants, the thorax is small, and the external force is transmitted to deep parts of the body with pinpoint accuracy, resulting in caudate lobe injury. The caudate lobe injury may be caused by an injury morphology similar to an injury to the extrahepatic bile duct. In other words, the deceleration mechanism may have caused an external force in the vertical direction to shear the porta hepatis [11]. In our own case, in addition to a laceration of the caudate lobe centered on the porta hepatis, there was also a laceration along the hepatic sickle mesentery that did not reach the hepatic surface. The result was a retroperitoneal hematoma via the reticulum, and the direction of the external force may have been the key point. It is very possible that this could have been prevented if the child had been properly secured in the car seat.

Caudate lobe injury and inferior vena cava injury are classified as high-level retroperitoneal hematomas. These hematomas are located higher than those associated with pelvic fractures and are difficult to identify on abdominal ultrasonography. In particular, the threshold for contrast-enhanced CT in children may require sedation and is higher than that in adults [12]. In addition, minor caudate lobe injuries and injuries to the inferior vena cava may be easily overlooked because they may not be evident on initial CT.

Recognizing slight circulatory changes at an early stage and being aware of the possibility of high-level retroperitoneal hematoma is important. In this case, the patient was on aspirin after surgery for TAPVC, and while the retroperitoneal hemorrhage could usually be stopped by the tamponade effect, the hemorrhage was slow but life-threatening in this instance. 

## Conclusions

Caudate lobe injuries in children are very rare, and their clinical presentation may be easily underestimated because of the initial circulatory stability due to the presence of a high retroperitoneal hematoma, which may be overlooked due to the high threshold of contrast-enhanced CT in children. The treating physician should keep in mind that once a circulatory disturbance occurs, the anatomically large vascular structures are in close proximity, making vascular injury more likely and necessitating surgical intervention.

## References

[1] Eichelberger M, Mooney D (2003). Abdominal trauma in operative pediatric surgery. Pediatrics International.

[2] Kepertis C, Zavitsanakis A, Filippopoulos A, Kallergis K (2008). Liver trauma in children: our experience. J Indian Assoc Pediatr Surg.

[3] Coccolini F, Coimbra R, Ordonez C (2020). Liver trauma: WSES 2020 guidelines. World J Emerg Surg.

[4] Dodds WJ, Erickson SJ, Taylor AJ, Lawson TL, Stewart ET (1990). Caudate lobe of the liver: anatomy, embryology, and pathology. AJR Am J Roentgenol.

[5] Abdalla EK, Vauthey JN, Couinaud C (2002). The caudate lobe of the liver: implications of embryology and anatomy for surgery. Surg Oncol Clin N Am.

[6] Brody AS, Kaufman RA, Kirks DR (1988). Isolated caudate lobe liver injury in a child: CT demonstration. J Comput Assist Tomogr.

[7] Romano L, Giovine S, Guidi G, Tortora G, Cinque T, Romano S (2004). Hepatic trauma: CT findings and considerations based on our experience in emergency diagnostic imaging. Eur J Radiol.

[8] Sacchi M, Scala T, Di Gaetano G (2000). Management of caudate lobe injury. Case report. Chir Ital.

[9] Iwasaki Y, Tani I, Nakajima Y (2001). Lesser sac hematoma as a sign of rupture of hepatocellular carcinoma in the caudate lobe. Eur Radiol.

[10] Nakao A, Matsuda T, Koguchi K (2000). Spontaneous rupture of hepatocellular carcinoma of the caudate lobe. Anticancer Res.

[11] Ramia JM, Gutiérrez G, Garrote D, Mansilla A, Villar J, Ferron JA (2005). Isolated extrahepatic bile duct rupture in blunt abdominal trauma. Am J Emerg Med.

[12] Ramalho CE, Bretas PM, Schvartsman C, Reis AG (2017). Sedation and analgesia for procedures in the pediatric emergency room. J Pediatr.

